# Response of sorghum genotypes to anthracnose (*Colletotrichum sublineolum*) resistance under field conditions in eastern Ethiopia

**DOI:** 10.1371/journal.pone.0316016

**Published:** 2024-12-30

**Authors:** Girmay Aragaw, Habtamu Terefe

**Affiliations:** 1 Department of Plant Science, Debre Tabor University, Debre Tabore, Ethiopia; 2 School of Plant Sciences, Haramaya University, Dire Dawa, Ethiopia; Lasbela University of Agriculture Water and Marine Sciences, PAKISTAN

## Abstract

Sorghum anthracnose is one of the major diseases that have negative impacts on sorghum production in Ethiopia. This study was conducted to evaluate the resistance response of sorghum genotypes against sorghum anthracnose and to determine yield performances of sorghum genotypes under field conditions in two consecutive cropping years. Forty-nine sorghum genotypes were tested for their reactions to anthracnose in the East and West Hararghe Zones, East Ethiopia, during the 2017 and 2018 main cropping seasons. The design of the experiment was laid out in triple lattice square design with three replications. Anthracnose severity was assessed on 16 randomly selected and pre-tagged sorghum plants in the middle two rows of each plot. The eight subsequent times of severity assessments were used to evaluate the response of the genotypes to sorghum anthracnose. The ranges of the mean severity of anthracnose in 2017 and 2018 were 35 to 91% and 38 to 93%, respectively. In 2017 and 2018, the area under disease progress curve varied from 1744 to 3865% of days and from 2354 to 4908% of days, respectively. During the two growing seasons, the genotype ETSL 101469 displayed the highest anthracnose severity, followed by BTX-623. In both experimental years, anthracnose had very strong negative relationships with grain yield and thousand seed weight. The current study demonstrated how Ethiopian sorghum genotypes affect anthracnose development in the field conditions. Throughout the two experimental years, the sorghum genotypes ETSL 100335, ETSL 100395, ETSL 10474, ETSL 100523, ETSL 100498, ETSL 100989, ETSL 100597, and ETSL 101000 continuously exhibited lower disease levels than the other genotypes. Therefore, it is advised to use these genotypes as alternate sources of sorghum anthracnose resistance. Nonetheless, further research across location is necessary to validate their anthracnose resistance in a variety of agro-ecologies.

## Introduction

Sorghum (*Sorghum bicolor* L. Moench) is farmed for a variety of purposes in both tropical and temperate climates of the world [1]. It is a source of fiber, fuel, food, and feed for diverse environments and production systems. Moreover, it is a crop that is viable for both humans and livestock in hot and arid climates worldwide [2]. After maize, rice, wheat, and barley, it is the world’s fifth most significant cereal crop [3]. After Sudan, Ethiopia is the second-most sorghum-growing nation in Eastern and Southern Africa [4]. Sorghum is the third important cereal crop next to teff and maize, took up to 14.21% (1,828,182.49 ha) of the grain crop area coverage in Ethiopia. In the country, up to 5.27 million tons of the cereal grain yield is contributed by sorghum [5]. Moreover, it covered 4.3 million tons of cereal production in eastern parts of Ethiopia, which is lower than national production [5].

The main cause of low output is because of many of the infectious and non-infectious production restrictions that seriously hinder sorghum productivity, with anthracnose posing the biggest challenge to growers [6]. One of the main limiting biotic factors in sorghum-producing nations worldwide, including Ethiopia, is sorghum anthracnose [7,8]. Due to this, in Ethiopia mean crop productivity is lower (2.80 t ha-1) [5] than the sorghum production potential (> 3.0 t ha-1) [9], despite being higher than the world’s average of 1.60 t ha-1 [10]. Likewise, sorghum average productivity in the research area is deficit as compared to the national mean grain yield (2.68 t ha-1) [5].

Sorghum anthracnose (*Colletotrichum sublineolum)*, one of the most destructive diseases that result in a complete loss of the crop [2,11]. It is a widespread disease throughout the world’s sorghum-growing regions [12,13]. However, tropical and subtropical regions with warm and humid climates have extreme severe disease [7]. Anthracnose is widely reported in East Africa, including Kenya, Uganda, and Ethiopia [14**]**. The pathogen can overwinter in soils through plant residues and seeds **[**15,16**].** Host resistance, pathogenic variability, and humid environmental conditions are among the disease epidemic elements that cause variation of anthracnose symptoms from elliptical red spots to elongated lesions with abundant acervuli [17]. Even though different epidemic elements affect this foliar disease, rainfall is a major factor and strongly influences anthracnose symptom development [18]. Sorghum leaves, stems, panicles, and grains are known to be attached by foliar anthracnose symptoms [19]. However, its effects are more severe on the leaf, where they reduce photosynthesis and, as a result, yield [20]. Yield losses of more than 50% may occur under severe epidemic conditions [7,21] also reported that yield reduction in Mali as a result of anthracnose ranged from 44 to 67%. Sorghum anthracnose in Ethiopia reduces yields on susceptible cultivars by 50–70% [11], which implies the designing and implementation of effective management practices.

Various management strategies have been tried, with varying degrees of success, to reduce the consequences of sorghum anthracnose. These included changing of planting dates, removing crop residues, use of host resistance, and applying fungicides [21,22]. Cultural practices are inexpensive and environmentally friendly but may be ineffective [17]. In addition, pesticide control is expensive and environmentally hazardous due to its pollution effect and fungicide resistance development [23]. Conversely, disease resistance variety is the most practical and cost-effective way to stabilize crop productivity [7]. Furthermore, developing disease-resistant varieties through breeding is environmentally viable, economical, and advantageous for small-holder growers [24]. Therefore, in order to find effective and sustainable ways to control sorghum anthracnose, scholars must look for potential sources of resistance and breed for disease resistance [25]. Genetic variability for anthracnose resistance may be present in the Ethiopian sorghum germplasm collection, which has proven to be a useful source of genetic variants [26]. Moreover, Ethiopian germplasm may be a significant source of anthracnose resistance [27]. Research on evaluating Ethiopian sorghum land races to identify anthracnose-resistant genotypes is currently ongoing, despite the fact that Ethiopia is one of the centers of genetic diversity for sorghum and provides germplasm for host-plant resistance to anthracnose. Therefore, this study was conducted to evaluate the resistance response of sorghum genotypes against sorghum anthracnose and to determine yield performances of sorghum genotypes under field conditions in two consecutive cropping years.

## Materials and methods

### Description of the study site

The research was carried out at Hirna, crop research sub-sit of Haramaya University, in West Hararghe Zone of eastern Ethiopia, during the 2017 and 2018 main cropping seasons. Hirna research center is located at 41° 4’ East longitude, 9° 12’ North latitude, and at elevation of 1870 m.a.s.l. The site receives a mean minimum rain fall of 990 and maximum 1010 mm, with an average temperature of 24°C. The soil of Hirna is vertisol with organic carbon content of 1.75%, total nitrogen content of 0.18%, available phosphorus content of 32 mg kg soil^-1^, exchangeable potassium content of 0.68 cmolc kg soil^-1^, pH of 7.09 and sand, silt and clay contents of 27, 28 and 45%, respectively [28].

### Experimental materials

Forty-eight sorghum genotypes were collected from the Sorghum and Millet Innovation Lab (SMIL) core collection, which were maintained by the Melkassa Agricultural Research Center and Haramaya University in collaboration with the SMIL project. Sorghum genotypes examined in this study were chosen according to their field performances. Genotypes that showed good agronomic characteristics, such as yield performance on the previous cropping year in Haramaya University, were selected and tested for their resistance reactions to sorghum anthracnose in the 2017 and 2018 main cropping seasons. One universally susceptible check (BTX623) [29] was utilized for two study seasons in addition to the 48 sorghum genotypes mentioned above. It was also used as a spreader row on all sides of the field.

### Treatments, experimental design and procedures

Planting was made on 10 May 2017 and 27 April 2018 main cropping seasons. The experiment was laid out in triple lattice design (TLD) with three replications. The two-year experiments were conducted in a natural field condition. Plots of 3 x 3 m (9 m^2^) with planting spaces of 75 x 20 cm were used and each plot consisted of four rows. Spacing between sub-blocks was 1.5 m and 2 m gang ways were left between replications. In each plot, 60 plants were sown, and each row had 15 sorghum crops. All agronomic practices were applied as per recommendations for better growing of sorghum. Fertilizers at a rate of 100 kg ha⁻^1^ of DAP and UREA were applied. All DAP was applied at planting, while urea was applied twice, half at planting and the remaining half at knee height of the sorghum. Cultivation and weeding were carried out four times after planting.

### Data collection

#### Disease assessment

Anthracnose severity was determined as the percentage of leaf area attacked by foliar anthracnose on 16 randomly chosen plants in the middle two rows of each plot. The severity of anthracnose was measured for each cropping year at the beginning of 80 days after planting for eight subsequent times and at 10-day intervals, which coincided with the onset of symptoms of the disease on the BTX-623 (universally susceptible check) sorghum genotypes. Severity was assessed by using 1–5 disease rating scale suggested by [30]; where, 1 = no visible symptoms, presence of chlorotic flecks; 2 = 1–10% leaf area covered with hypersensitive lesions without acervuli; 3 = 11−25% leaf area covered with hypersensitive and restricted lesions without acervuli; 4 = 26−50% leaf area covered by coalescing necrotic lesions with acervuli and 5 = >50% leaf area covered with necrotic lesions and acervuli. Severity scales were categorized into resistance groups in which severity scale of 1 was considered as highly resistant while scales 2, 3, 4 and 5 were regarded as resistant, moderately resistant, susceptible and highly susceptible, respectively. Using the formula proposed by [31], severity ratings were transformed into percent severity index (PSI) for analysis.


$$PSI=\frac{Sum\;of\;numerical\;ratings}{No.of\;plants\;scored\;x\;maximum\;score\;on\;scale}x\;100$$

Using the [32] suggested method; the area under the disease progress curve (AUDPC) was computed from the severity data as follows:

$$AUDPC=\sum_{i=1}^{n-1}\left[\frac{\left(X_{i}+X_{i+1}\right)}{2}\right](t_{i+1}-t_{1})$$

Where, Xi = percentage of disease severity at i^th^ assessment; ti = time of the i^th^ assessment in days from the first assessment date and n = total number of disease assessments.

#### Yield and thousand seed weight

Each plot’s middle two rows were used to gather grain yield. Harvesting was done manually and plants were cut at their base, placed in their respective plots and allowed to dry. The dried sorghums were then threshed, and the total grain obtained from each plot was adjusted to 12.5% grain moisture content and converted to t ha^-1^. Thousand seed weight was determined from composite samples taken from each plot of the harvested grain yield. The 1000 seeds were counted using a seed counter, and the weight was determined by an electronic sensitive balance.

#### Meteorological data

**Fig 1:**
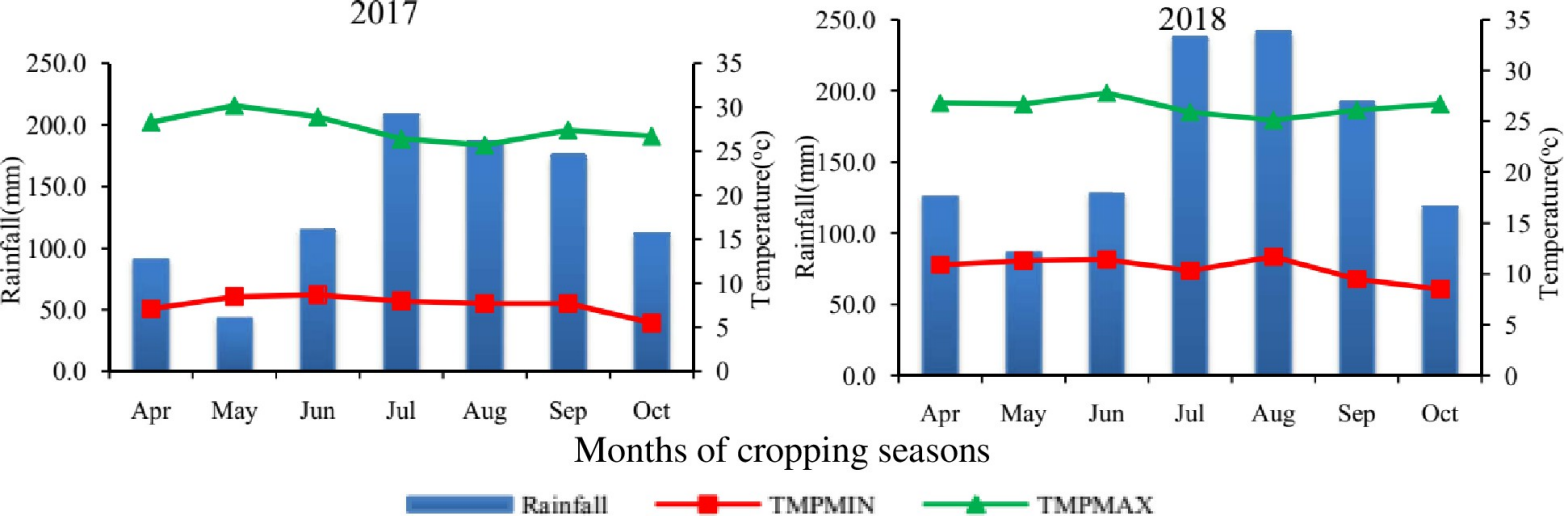
Monthly average rainfall (mm), maximum and minimum temperatures (oC) at Hirna, eastern Ethiopia, during 2017 and 2018 main cropping seasons.

The national meteorological agency provided the monthly minimum and maximum temperatures (°C) and rainfall (mm) for the 2017 and 2018 main cropping seasons of the research area. The study region experienced monthly rainfall ranging from 43.2 to 208.5 mm in 2017 and from 86.3 to 241.6 mm in 2018 cropping years. Compared to the 2018 cropping season, there was less rainfall in year 2017 (Fig 1). In 2017, the daily minimum and maximum temperatures fluctuated from 5.5 to 8.7 ^**o**^**C and 25.7 to 30.2** ^**o**^**C**, respectively. In the year 2018, the daily minimum and maximum temperatures varied from 8.5 to 11.7°C and 25.1 to 27.8°C, respectively. Overall, it was discovered that the climate in the area was suitable for the development of sorghum anthracnose epidemics.

#### Data analyses

For each year, SAS software version 9.4 was used to conduct analysis of variance (ANOVA) on the following variables: disease severity, AUDPC, yield, and thousand seed weight. Duncan’s Multiple Range Test (DMRT) was used to compare means at the 5% significance level. To determine the disease progress rate from the linear regression, a Logistic model, ln[(y/1-y)], [33] was used to estimate the disease progression after transforming severity data. The transformed severity data were regressed over time (days after planting) to determine the disease progress rate. Regression was computed using Minitab (Release 18.0). The two seasons were considered as different because of heterogeneity of variances as tested using Bartlett’s test [34] and the F-test was significant for most of the parameters studied. Thus, data were not combined for analysis. The Pearson’s correlation coefficients between pairs of yield traits with disease parameters were tested for their significance using SAS software.

## Results

### Anthracnose severity

Regardless of the experimental years, none of the tested sorghum genotypes were immune to sorghum anthracnose. However, in this study, the responses of Ethiopian sorghum genotypes were different and significantly (P <0.01) variable for disease severity during both experimental years. The overall impact of anthracnose in 2018 was higher than the 2017 cropping season (Table 1). Moreover, during the 2018 evaluation year, up to 17.63% mean anthracnose severity increments were observed among tested genotypes.

**Table 1:** Percent severity index (%) and area under the disease progress curve (%-days) of anthracnose on 49 sorghum genotypes tested in eastern Ethiopia during the 2017 and 2018 main cropping seasons.

Genotype	PSI^1^		AUDPC^2^		Resistance reaction class^3^	
	2017	2018	2017	2018	2017	2018
ETSL 100588	69.34cd	70.50^b-f^	2797.5^c-e^	3462.9^e-h^	S	S
ETSL 100709	48.22^o-r^	53.83^k-q^	2251.8^j-p^	2914.1^n-p^	R	MR
ETSL 101476	67.62^c-e^	69.69^c-g^	2981.9^b-d^	3629.0^de^	MR	S
IS 38394	66.64^c-f^	70.64^b-f^	2958.6^b-d^	3395.5^e-i^	MR	S
ETSL 100368	45.63^p-s^	54.00^k-q^	2043.9^o-s^	2909.3^n-p^	R	MR
ETSL 100252	48.50^o-r^	54.81^j-p^	2069.2^n-s^	2988.6^k-p^	R	MR
ETSL 101469	91.23^a^	93.19^a^	3865.1^a^	4907.5^a^	HS	HS
ETSL 101143	60.71^g-j^	65.72^d-i^	2695.1^d-h^	3550.6^d-f^	MR	MR
ETSL 100518	58.69^h-k^	61.89^f-l^	2725.7^d-g^	3054.3^j-p^	MR	MR
ETSL 100523	39.16^t-v^	48.36^o-s^	1899.8^q-t^	2796.6^o-r^	R	R
IS 25542	56.81^i-l^	68.94^c-h^	2363.8^i-n^	3236.8^h-l^	MR	S
ETSL 100498	38.78^t-v^	45.06^q-t^	1981.0^o-t^	2560.8^rs^	R	R
ETSL 101042	55.54^j-n^	70.69^b-f^	2261.4^j-o^	3991.4^c^	MR	S
ETSL 100335	34.78^v^	38.16^t^	1743.5^t^	2353.9^s^	R	R
ETSL 101191	65.63^d-g^	70.28^b-f^	2645.3^e-i^	3620.8^de^	MR	S
ETSL 101470	46.88^p-s^	54.03^k-q^	1996.9^o-t^	2959.7^l-p^	R	MR
ETSL 100395	36.27^uv^	42.33^st^	1879.3^r-t^	2498.9^s^	R	R
ETSL 101038	46.48^p-s^	57.75^i-o^	2170.9^k-r^	2952.0^l-p^	R	MR
ETSL 101522	43.39^r-t^	53.92^k-q^	2090.0^n-s^	3157.3^i-n^	R	MR
ETSL 100989	41.38^s-u^	47.14^p-s^	2063.5^n-s^	2585.5^q-s^	R	R
Chelenko	48.35^o-r^	55.78^j-p^	2004.7^o-t^	2987.5^k-p^	R	MR
ETSL 100959	53.50^k-o^	58.64^i-n^	2181.1^k-r^	3238.5^h-l^	MR	MR
ETSL 100470	45.50^q-s^	51.97^m-r^	2071.7^n-s^	2970.9^k-p^	R	MR
IS 38282	56.30^i-m^	61.69^f-l^	2183.8^k-r^	3217.5^h-m^	MR	MR
ETSL 101597	50.93^l-q^	57.53^i-o^	2498.0^f-j^	3152.7^i-n^	MR	MR
IS 38401	61.69^f-i^	65.72^d-i^	2468.6^g-k^	3479.0^e-h^	MR	MR
ETSL 100597	43.47^r-t^	49.67^n-s^	1955.0^o-t^	2803.4^o-r^	R	R
ETSL 101687	72.18^bc^	75.64^bc^	2808.1^c-e^	3806.9^cd^	S	S
ETSL 101050	49.87^o-n^	59.64^h-m^	2190.9^k-q^	2926.1^m-p^	MR	MR
ETSL 101285	75.76^b^	79.28^b^	3128.4^b^	3909.3c	S	S
BTX-623	88.12^a^	91.47^a^	3138.5^b^	4518.3^b^	S	HS
ETSL 101373	65.35^d-g^	73.81^b-d^	2765.5^c-f^	4038.2^c^	MR	S
ETSL 101000	41.93^st^	51.00^m-s^	1942.8^p-t^	2927.5^m-p^	R	R
AL-70	63.13^gfhe^	68.58^c-h^	2415.7^h-m^	3383.5^e-i^	MR	MR
ETSL 101052	50.71^m-q^	59.11^i-n^	2468.8^g-k^	3387.6^e-i^	MR	MR
ETSL 100328	56.20^i-m^	59.97^h-m^	2743.1^d-g^	3260.3^g-k^	MR	MR
ETSL 101474	36.11^uv^	43.00^r-t^	1807.1^st^	2463.4^s^	R	R
ETSL 101482	53.31^k-o^	70.94^b-f^	2634.0^e-i^	3556.4^d-f^	MR	S
ETSL 101212	68.90^cd^	72.39^b-e^	3043.0^bc^	3493.5^e-h^	S	S
IS 38276	54.93^j-n^	59.69^h-m^	2054.9^o-s^	3080.5^j-p^	MR	MR
IS 38348	56.73^i-m^	60.67^g-m^	2134.9^l-r^	3085.5^j-o^	MR	MR
ETSL 100309	47.07^p-s^	54.64^k-p^	2249.7^j-p^	3011.1^j-p^	R	MR
ETSL 101309	51.72^l-p^	56.05^j-p^	2425.5^h-l^	2885.4^n-p^	MR	MR
ETSL 101008	45.78^p-s^	52.75^l-q^	2203.7^j-q^	2947.2^l-p^	R	MR
ETSL 100579	47.03^p-s^	58.00^i-n^	1963.8^o-t^	2784.2^p-r^	R	MR
ETSL 101048	55.21^j-n^	64.33^e-j^	2093.7^n-s^	3534.6^d-g^	MR	MR
ETSL 101743	58.14^h-k^	62.54^f-k^	2749.6^d-g^	3280.7f^g-j^	MR	MR
ETSL 101523	45.27^q-s^	52.53^l-q^	2117.7^m-r^	2958.5^l-p^	R	MR
ETSL 100815	49.63^o-n^	54.14^k-q^	2109.8^n-s^	2838.0^o-q^	MR	MR
Mean	54.17	60.66	2368.09	3213.30		
CV (%)	5.78	7.96	6.52	4.64		
P value (%)	40.96**	14.34**	20.72**	28.73**		

^1^PSI = Percent severity index. ^2^AUDPC = Area under disease progress curve of sorghum anthracnose. ^3^Resistance reaction class: R = resistant (scale 2); MR = moderately resistant (scale 3); S = susceptible (scale 4) and HS = highly susceptible (scale 5). CV (%) = Coefficient of variation

** = highly significant at P≤0.0001 probability level, mean values with the same letter(s) within a column are not significantly different from each other at P<0.05 probability level.

In the year 2017, the variations of average sorghum anthracnose severity were between 35 and 91%. Genotype ETSL101469 results in the highest (91.2%) sorghum anthracnose severity than other tested materials, which was followed by the universally susceptible check BTX-623 (88.1%). On the other hand, the lower anthracnose severities (34.8%, 36.1%, and 36.3%) were recorded on genotypes ETSL100335, ETSL101474, and ETSL100395, respectively. During the 2018 experimental year, the highest (93.2%) disease severity was also recorded on genotype ETSL101469 as compared to the other genotypes, including the susceptible check BTX-623, which had 91.5% mean anthracnose. Genotype ETSL100335 recorded the lowest (38.2%) disease severity than other genotypes evaluated at field condition (Table 1). In general, sorghum anthracnose showed continues rate of disease progresses starting from the onset to the final anthracnose severity masseurs in both growing seasons.

### Area under disease progress curve (AUDPC)

The disease severity records were used to calculate the area under disease progress curve (%-days), which revealed a highly significant (P<0.01) difference between the sorghum genotypes that were examined in both cropping years. All studied genotypes had higher AUDPC values calculated for the 2018 cropping season compared to the 2017 (Table 1). The AUDPC values for 2017 cropping season ranged from 1744% to 3865%-days. The genotype ETSL 101469 had the highest AUDPC value, whereas the genotype ETSL 100335 had the lowest value, followed by ETSL 101474 (1807.1% days) and ETSL 100395 (1879.3% days). On the other hand, throughout the 2018 growing season, AUDPC values varied from 2354% on ETSL 100335 to 4908%-days on the genotype ETSL 101469 (Table 1). The findings of the two-year study showed that the tested sorghum genotypes responded differently to anthracnose infections that occur naturally in eastern Ethiopia.

### Host reaction to sorghum anthracnose

The sorghum genotypes tested in this study were categorized into several severity classes according to the final anthracnose severity, which was measured 150 days after sowing (Table 1). Eight genotypes were found to be resistant during the 2018 cropping season, compared to the 20 (40.3%) that were rated as resistant in 2017. Genotypes ETSL 100335, ETSL 100395, ETSL 101474, ETSL 100523, ETSL 100498, ETSL 100989, ETSL 100597 and ETSL 101000 were consistently evaluated as resistant. In the 2017 and 2018 cropping seasons, respectively, twenty-three (46.9%) and twenty-eight (57.1%) sorghum genotypes had shown a moderately resistant response to anthracnose. About ten percent of the materials evaluated in 2017 and eleven (22.5%) genotypes in the 2018 cropping year exhibited susceptible reactions. Additionally, in the evaluation years 2017 and 2018, respectively, one and two genotypes demonstrated a very susceptible response to anthracnose under natural infection (Table 1). Variation in reaction to sorghum anthracnose might be the presence of variable genetic makeup among the tested sorghum genotypes. This reveals that these genotypes could be utilized in the breeding program for anthracnose-resistant sorghum genotype development.

### Disease progress rate (r)

In both cropping years, there were differences in the disease progression rates across the genotypes of sorghum that were investigated (Table 2). Anthracnose progression rate in 2017 planting year ranged from 0.0084 to 0.0463 units day^**-1**^. Anthracnose progress was the fastest on genotype ETSL 101469, while it was the slowest on ETSL 100395 genotype (Table 2). The disease progress rate ranged from 0.0107 to 0.0479 units day^**-1**^ in the 2018 growing season. The faster (0.0479 and 0.0470) rates of disease development were recorded on BTX-623 and ETSL 101469 genotypes, respectively than others. However, genotypes ETSL 100335 (0.0107 units day^**-1**^), ETSL 100395 (0.0122 units day^-1^), and ETSL 101474 (0.0137 units day^**-1**^) showed the slower rates of anthracnose development (Table 2). The 2018 cropping year saw a comparatively quicker spread of the anthracnose outbreak than in 2017.

**Table 2:** Mean initial (PSI_i_) and final (PSI_f_) severity and parameter estimates of sorghum anthracnose on 49 sorghum genotypes at Hirna, eastern Ethiopia, during the 2017 and 2018 cropping years.

Genotype	Percent severity^1^				Disease progress		R^2^ (%)^2^	
	PSI _i_	PSI _f_	PSI _i_	PSI _f_	rate (units day^-1^)			
	2017		2018		2017	2018	2017	2018
ETSL 100588	23.33	69.34	22.78	70.50	0.0225	0.0268	80.47	93.61
ETSL 100709	22.50	48.22	24.03	53.83	0.0149	0.0193	78.38	92.40
ETSL 101476	23.49	67.62	24.72	69.69	0.0237	0.0237	91.29	86.81
IS 38394	23.89	66.64	24.31	70.64	0.0237	0.0257	93.12	86.77
ETSL 100368	20.00	45.63	21.81	54.00	0.0142	0.0211	83.38	87.34
ETSL 100252	21.81	48.50	21.67	54.81	0.0146	0.0201	79.10	88.61
ETSL 101469	25.42	91.23	33.06	93.19	0.0463	0.0470	81.72	95.07
ETSL 101143	23.06	60.71	23.89	65.72	0.0204	0.0248	91.43	89.30
ETSL 100518	23.60	58.69	23.61	61.89	0.0183	0.0235	88.10	93.15
ETSL 100523	20.00	39.16	21.53	48.36	0.0121	0.0168	81.32	80.74
IS 25542	21.39	56.81	22.92	68.94	0.0197	0.0269	80.02	89.05
ETSL 100498	21.53	38.78	23.06	45.06	0.0107	0.0152	76.55	90.31
ETSL 101042	20.83	55.54	30.56	70.69	0.0182	0.0223	81.31	79.52
ETSL 100335	20.00	34.78	22.36	38.16	0.0087	0.0107	77.34	77.07
ETSL 101191	22.22	65.63	23.75	70.28	0.0213	0.0275	82.35	95.10
ETSL 101470	20.28	46.88	23.33	54.03	0.0153	0.0203	83.46	85.29
ETSL 100395	20.83	36.27	21.94	42.33	0.0084	0.0122	78.55	74.69
ETSL 101038	20.42	46.48	22.64	57.75	0.0154	0.0214	89.22	85.97
ETSL 101522	22.64	43.39	22.93	53.92	0.0117	0.0164	84.24	86.21
ETSL 100989	22.08	41.38	22.92	47.14	0.0117	0.0160	78.56	90.71
Chelenko	20.56	48.35	23.06	55.78	0.0143	0.0215	75.22	92.40
ETSL 100959	21.49	53.50	26.53	58.64	0.0166	0.0170	76.36	87.37
ETSL 100470	22.10	45.50	22.92	51.97	0.0125	0.0170	72.55	83.30
IS 38282	20.00	56.30	22.78	61.69	0.0199	0.0247	85.99	90.51
ETSL 101597	22.36	50.93	22.64	57.53	0.0142	0.0216	84.34	86.91
IS 38401	20.83	61.69	25.69	65.72	0.0214	0.0239	82.96	92.06
ETSL 100597	20.69	43.47	22.92	49.67	0.0131	0.0158	83.49	81.49
ETSL 101687	22.78	72.18	24.72	75.64	0.0239	0.0287	82.46	90.29
ETSL 101050	21.81	49.87	22.5	59.64	0.0163	0.0217	87.90	89.85
ETSL 101285	24.44	75.76	23.89	79.28	0.0262	0.0330	84.71	94.17
BTX-623	22.92	88.12	25.69	91.47	0.0397	0.0479	84.69	93.35
ETSL 101373	21.39	65.35	25.28	73.81	0.0256	0.0312	92.64	88.37
ETSL 101000	20.69	41.93	23.89	51.00	0.0113	0.0175	76.49	81.73
AL-70	20.83	63.13	23.47	68.58	0.0235	0.0275	89.49	96.43
ETSL 101052	21.39	50.71	23.33	59.11	0.0155	0.0215	80.05	81.59
ETSL 100328	23.92	56.20	24.44	59.97	0.0164	0.0195	89.28	88.31
ETSL 101474	20.56	36.11	21.67	43.00	0.0098	0.0137	76.35	86.91
ETSL 101482	22.64	53.31	24.03	70.94	0.0146	0.0288	79.45	79.01
ETSL 101212	23.89	68.90	24.31	72.39	0.0225	0.0266	88.05	93.68
IS 38276	20.14	54.93	21.94	59.69	0.0177	0.0232	74.10	92.88
IS 38348	20.28	56.73	22.08	60.67	0.0194	0.0234	83.25	93.36
ETSL 100309	21.51	47.07	23.06	54.64	0.0147	0.0194	90.99	88.48
ETSL 101309	22.64	51.72	22.22	56.05	0.0149	0.0200	77.10	91.08
ETSL 101008	21.11	45.78	21.94	52.75	0.0161	0.0190	84.88	82.90
ETSL 100579	21.11	47.03	20.97	58.00	0.0134	0.0209	75.98	76.51
ETSL 101048	20.28	55.21	26.94	64.33	0.0171	0.0230	70.11	80.39
ETSL 101743	23.43	58.14	24.51	62.54	0.0196	0.0227	89.26	91.92
ETSL 101523	20.56	45.27	23.61	52.53	0.0146	0.0185	82.30	87.05
ETSL 100815	21.25	49.63	22.5	54.14	0.0162	0.0204	84.02	92.23

^1^Initial and final disease severity (PSI) of sorghum anthracnose were recorded at 80 and 150 days after planting, respectively.

^2^Coefficient of determination of the Logistic epidemiological model. Parameter estimates are obtained from a linear regression of ln(y/(1-y)) disease severity proportions with days after planting.

### Yield and thousand seed weight

Highly significant (P<0.01) differences were observed in grain yield and thousand seed weight across the studied sorghum genotypes (Table 3). Weight of thousand seeds varied in the 2017 planting year from 17.8 g on genotype ETSL 101373 to 40 g on genotype ETSL 101008. While the weight of 1000 seeds in the 2018 study year ranged from 16.1 to 40.6 g on genotypes ETSL 1013773 and ETSL 100309, respectively. In the 2017 cropping year, the genotype with the resistant reaction (ETSL 100335) produced the highest grain yield (10.6 t ha^-1^), the genotypes with the lowest yields (3.18 and 3.44 t ha^-1^) were BTX-623 and ETSL 101373, respectively. The best grain yield, 8.3 t ha^-1^ was observed in the 2018 cropping season from genotype ETSL 100335. However, the genotypes with the lowest grain yields, 2.7, 3.16, and 3.67 t ha^-1^ were BTX-623, ETSL 101373, and ETSL 101469, in that order (Table 3). This result indicated that the tested genotypes were different in their yield performance, and this might be due to genetic variation, the effect of foliar anthracnose, or environmental factors. In addition, the highest yield of the present study was found in 2017 compared to the 2018 experimental year. This is because of the effect of high sorghum anthracnose intensity pressure in 2018 that resulted in a poor grain-filling of the genotypes.

**Table 3:** Thousand seed weight and grain yield of sorghum genotypes evaluated at Hirna, eastern Ethiopia, during the 2017 and 2018 main cropping seasons.

Genotype	Thousand seed weight (g)		Grain yield (t ha^-1^)	
	2017	2018	2017	2018
ETSL 100588	33.03^b-i^	30.3^d-j^	5.62^g-m^	4.64^g-l^
ETSL 100709	26.33^k-q^	27.13^h-o^	7.29b^c-j^	4.75^g-k^
ETSL 101476	24.97^n-q^	24.3^n-p^	4.89^j-m^	4.79^g-k^
IS 38394	34.67^b-f^	28.9^d-l^	6.58^d-k^	4.26^j-l^
ETSL 100368	35.0^b-e^	30.73^c-i^	8.27^a-f^	8.01^ab^
ETSL 100252	30.47^d-l^	27.37^g-o^	5.51^h-m^	4.64^g-l^
ETSL 101469	24.97^n-q^	24.57^m-p^	4.16^k-m^	3.67^kl^
ETSL 101143	29.47^g-o^	26.07^k-o^	6.07^d-k^	4.81^f-k^
ETSL 100518	33.2^b-h^	27.47^g-o^	6.71^d-k^	5.07^e-k^
ETSL 100523	31.13^d-^l	32.8^cd^	8.36^a-e^	8.07^ab^
IS 25542	25.37^m-q^	23.43^op^	6.56^d-k^	5.51^c-k^
ETSL 100498	30.07^e-m^	27.5^g-o^	6.0^e-l^	6.32^b-i^
ETSL 101042	27.9^j-p^	25.2^l-p^	7.2^c-j^	5.26^d-k^
ETSL 100335	36.4^a-c^	32.0^c-f^	10.6^a^	8.32^a^
ETSL 101191	26.37^k-q^	27.33^g-o^	4.92^j-m^	4.94^f-k^
ETSL 101470	35.07^b-d^	32.3^c-e^	8.4^a-e^	5.25^d-k^
ETSL 100395	30.87^d-l^	29.9^d-k^	7.8^b-i^	7.14^a-d^
ETSL 101038	29.73^f-n^	28.8^d-l^	9.67^a-c^	5.75^c-j^
ETSL 101522	27.97^j-p^	27.93^f-n^	6.71^d-k^	4.48^h-l^
ETSL 100989	30.94^d-l^	28.6^e-m^	6.78^d-j^	6.38^b-h^
Chelenko	29.4^g-o^	29.17^d-l^	9.8^ab^	6.79^a-f^
ETSL 100959	26.53^k-p^	25.57^l-p^	5.55^h-m^	4.87^f-k^
ETSL 100470	28.37^h-o^	25.43^l-p^	6.51^d-k^	4.23^j-l^
IS 38282	31.4d^e-k^	26.7^i-o^	5.89^e-l^	4.86^f-k^
ETSL 101597	33.5^b-g^	30.63^c-i^	6.64^d-k^	6.34^b-i^
IS 38401	28.1^i-o^	28.93^d-l^	6.59^d-k^	4.91^f-k^
ETSL 100597	28.9^g-o^	30.93^c-h^	7.42^b-j^	6.59^a-g^
ETSL 101687	23.1^pq^	25.87^k-o^	5.19^i-m^	3.89^j-l^
ETSL 101050	27.43^j-p^	28.67^e-m^	6.38^d-k^	4.96^f-k^
ETSL 101285	26.23^l-q^	27.0^h-o^	5.73^f-m^	4.63^g-l^
BTX-623	21.73^qr^	21.83^p^	3.44^lm^	2.70^l^
ETSL 101373	17.77^r^	16.1^q^	3.18^m^	3.16^kl^
ETSL 101000	27.8^j-p^	28.43^e-n^	5.33^h-m^	4.39^h-l^
AL-70	26.97^j-p^	26.4^j-o^	5.33^h-m^	3.76^j-l^
ETSL 101052	29.17^g-o^	31.37^c-g^	6.02^d-k^	5.08^e-k^
ETSL 100328	30.8^d-l^	30.93^c-h^	8.18^a-g^	5.39^d-k^
ETSL 101474	30.78^d-l^	30.33^d-j^	9.67^a-c^	7.02^a-e^
ETSL 101482	28.13^i-o^	28.4^e-n^	8.44^a-e^	5.24^d-k^
ETSL 101212	24.53^o-q^	26.633^i-o^	5.49^h-m^	4.36^i-l^
IS 38276	33.7^b-g^	26.93^h-o^	5.5^h-m^	4.62^g-l^
IS 38348	34.7^b-f^	28.83^d-l^	5.45^h-m^	4.37^i-l^
ETSL 100309	37.7^ab^	40.57^a^	8.44^a-e^	7.39^a-c^
ETSL 101309	31.83^c-j^	37.6^ab^	7.82^b-h^	5.74^c-j^
ETSL 101008	40.03^a^	32.32^c-e^	8.22^a-g^	5.62^c-k^
ETSL 100579	35.0^b-e^	34.47^bc^	8.29^a-f^	7.00^a-e^
ETSL 101048	30.87^d-l^	28.8^d-l^	5.33^h-m^	4.67^g-k^
ETSL 101743	26.83^j-p^	28.2^e-n^	5.53^h-m^	5.10^e-k^
ETSL 101523	28.97^g-o^	26.267^j-o^	6.82^d-j^	4.18^j-l^
ETSL 100815	30.0f^g-n^	28.03^f-n^	8.62^a-d^	6.52^a-g^
Mean	29.68	28.45	6.71	5.33
CV (%)	8.42	7.21	19.21	18.44
P value (%)	7.44**	8.74**	3.96**	3.95**

CV (%) = Coefficient of variation

** = highly significant at P≤0.0001 probability level, and mean values with the same letter(s) within a column are not significantly different from each other at P<0.05 probability level.

### Association of disease parameters and yield attributes

Studies on phenotype and genotype correlation can reveal the nature and strength of the relationship between any two pairs of characters. In both cropping years, there was a significant (P<0.01) and negative genotypic correlation coefficient between the parameters of the sorghum anthracnose and grain yield as well as thousand seed weight (Table 4). Disease severity showed negative relationships with grain yield (*r* = -0.69 and -0.70) and thousand seed weight (*r* = -0.50 and -0.51) in the 2017 and 2018 cropping seasons, respectively. Grain yield and AUDPC exhibited a highly negative genotypic connection (r = -0.57) in the 2018 and (r = -0.69) in the 2017 cropping years. However, genotypic correlation coefficient analysis results indicated that thousand seed weight had a highly significant (P<0.01) and positive association with grain yield in both cropping years. Disease severity also exhibited a highly and positively significant correlation with AUDPC in the two cropping years (Table 4).

**Table 4:** Genotypic (above diagonal) and phenotypic (below diagonal) correlation coefficients among yield and sorghum anthracnose parameters at Hirna, eastern Ethiopia, during the 2017 and 2018 main cropping seasons.

Parameter ^a^ Cropping year								
	2017				2018			
	FPSI	AUDPC	TSW	GY		FPSI	AUDPC	TSW	GY
FPSI	1	0.92**	-0.50**	-0.69**	1	0.95**	-0.51**	-0.70**
AUDPC	0.88**	1	-0.47**	-0.57**	0.90**	1	-0.55**	-0.69**
TSW	-0.45**	-0.41**	1	0.60**	-0.41**	-0.49**	1	0.64**
GY	-0.54**	-0.46**	0.48**	1	-0.53**	-0.54**	0.51**	1

^a^ FPSI = Final percent severity index (%), AUDPC = Area under disease progress curve (%-days), GY = Grain yield (t ha-^1^), and TSW = Thousand seed weight (g).

** = Very highly significant at P≤0.0001 probability level.

The results of the analysis of phenotypic correlation for disease severity and AUDPC revealed a strong and negative association (P<0.01) with grain yield and thousand seed weight in both cropping years (Table 4). In the 2017 and 2018 sowing time, there was a highly significant (P<0.01) positive association between thousand seed weight and grain yield. In the 2017 and 2018 research years, respectively, grain yield (r = -0.54 and -0.53) showed a highly significant and negative phenotypic correlation with anthracnose severity. Thousand seed weight (*r* = -0.45 in 2017) and (*r* = -0.41 in 2018) also negatively associated with disease severity. The extent of anthracnose impacts on sorghum production in the study areas and other relevant agro-ecologies was demonstrated by the negative genotypic and phenotypic relationships of disease parameters with grain yield and thousand seed weight.

## Discussion

Sorghum is grown all over the world; however, anthracnose significantly reduces its production and productivity. As chemical control of anthracnose is not practical or economically viable, the most desirable strategy for managing this disease has been thought to be host resistance [35]. Therefore, the aim of this experiment was to identify sources of stable genetic resistance sorghum genotype to control anthracnose in Eastern Ethiopia over the two cropping years. The evaluation findings showed that different Ethiopian sorghum genotypes responded differently to anthracnose in a natural infection. Throughout both experimental years, the 49 sorghum genotypes that were examined responded to anthracnose as resistant, moderately resistant, susceptible, and highly susceptible. Similarly, [8] reported that Ethiopian sorghum genotypes evaluated in southwestern parts of the country showed susceptible and resistant reactions to foliar anthracnose in two experimental years. [36] indicated that sorghum accessions during the two growing seasons in Texas responded to the anthracnose disease in a resistant and susceptible manner. Additionally, [37] observed that accessions to sorghum anthracnose reacted in a resistant, susceptible, and very susceptible manner. [30] also suggested that sorghum genotypes showed variable responses, with some genotypes exhibiting greater resistance than others. Therefore, breeders can use the differences in resistance reaction to the disease between genotypes to design a sustainable anthracnose control strategy, as these genotypes may have innate genetic resistance.

We discovered substantial differences in all anthracnose disease parameters (severity, area under the disease progress curve, disease progress rate, and disease progress curve) among the sorghum genotypes that were examined in this study. There were remarkable differences in the development of sorghum anthracnose between the genotypes. This might be the result of variations in the genetic resistance exhibited by the sorghum genotypes that have been assessed. Furthermore, this variability among the evaluated sorghum genotypes could be explained by the genetic inheritance and response of the genotypes and other factors like adaptability and the environmental factors. The varying responses of the sorghum genotypes to anthracnose suggested the possible roles that these genotypes could play in a future breeding program for anthracnose management. This result is consistent with the finding of [38], who showed varied sorghum genotype responses to foliar anthracnose. [39] also reported that a significant variance in sorghum genotypes resistance to anthracnose was discovered in Brazil. Accessions had a substantial impact on the anthracnose response, and there was diversity in the disease response amongst them [36]. In addition, [25] showed that sorghum accessions differed in how they responded to a natural *C*. *sublineolum* infection.

In the current work, over 16% and 32%, respectively, of the 49 sorghum genotypes assessed in the field during the two growing seasons demonstrated resistant and moderately resistant responses. Differences in reaction to foliar anthracnose observed among sorghum genotypes might inform the presence of variability in genetic makeup among the tested sorghum genotypes. This indicated that Ethiopian sorghum genotypes may be a significant source of anthracnose resistance to improve crop productivity. Similarly, [25] documented the response of sorghum materials over the course of the two experimental years, indicating moderate and resistance reaction to anthracnose. Of the 40 Ethiopian sorghum accessions evaluated, [40] also found that 20 of them were resistant to anthracnose. The results of [41] further demonstrated the widespread sources of disease resistance in Ethiopian sorghum accessions. After evaluating 132 sorghum landraces for foliar anthracnose, [42] identified 109 landraces that were resistant to the disease.

Based on this research, the 2018 cropping year showed more severe sorghum anthracnose than the 2017 cropping year. This outcome is comparable to that of [25], who noted differences in the course of sorghum anthracnose disease development during the two experimental years. According to [36] sorghum accessions have shown variations in disease response over the growing season. The variation in weather conditions and inoculum density differences across cropping years may be related to anthracnose variability with planting years. [35] Indicated that sorghum anthracnose development in the field is affected by high relative humidity, quantity of inoculum, high rainfall, and warm temperatures. It has been demonstrated that weather, especially rainfall, significantly influences how severe sorghum anthracnose is [11,41,43,44]. [18] also suggested rainfall frequency and accumulation play a crucial role in the development of sorghum anthracnose. It is most severe when there are prolonged cloudy, warm, humid, and damp weather conditions [45].

In the two cropping years of this study, there was a considerable variation in grain yield between the evaluated 49 sorghum genotypes. In addition, there was a highly significant negative correlation between anthracnose and sorghum grain yield in both years overall, with the year 2018 showing the greatest yield reduction compared to the cropping year 2017. This yield variation among genotypes and negative associations between disease and yield parameters might be due to the presence of genetic variation among the genotypes and high anthracnose disease intensity. Grain-filling was significantly impacted negatively by the sorghum anthracnose disease pressure, which causes significant yield losses in genotypes that are susceptible. In line with this finding, [8] reported that anthracnose caused a higher loss in grain yield in 2014 compared to the 2015 cropping year. He also demonstrated a strong negative correlation between anthracnose and grain yield in the two growing years. [46] also proposed that the poor development of panicle and grain was due to *C*. *sublineolum* conidia interfering with the passage of water and nutrients in the vascular tissues of plants. Moreover, [47] suggested a decrease in grain size is frequently linked to losses in grain yield caused by fungal infection.

## Conclusions

Enough genetic heterogeneity was found in the 49 sorghum genotypes evaluated for anthracnose disease response in this study. Different genotypes responded to sorghum anthracnose in different ways in the field, and environmental variables, particularly rainfall, and genotype resistance levels all had an impact on the severity of the disease. Tested sorghum genotypes exhibiting distinct characteristics can be utilized in breeding programs for sorghum by tapping into their genetic potential. The current research confirmed that Ethiopia has potential sources of resistant genes for the control of sorghum anthracnose. In both experimental years, the following genotypes: ETSL 100335, ETSL 100395, ETSL 101474, ETSL 100523, ETSL 100498, ETSL 100989, ETSL 100597, and ETSL 101000 demonstrated resistance response to sorghum anthracnose and produced good grain yield. Farmers in eastern Ethiopia can, therefore, employ the eight sorghum genotypes. Additionally, these genotypes can be used by breeders as a source of resistant genes in breeding programs to develop anthracnose resistant varieties. In this investigation, the reaction of sorghum genotypes to the disease displayed a considerable degree of diversity. However, because of genotype-environment interaction, morphological variation does not always reflect true genetic variation. Therefore, additional molecular research is required for more clarity of genetic variation of sorghum genotypes. Moreover, further study on over location should be conducted to validate their resistance to sorghum anthracnose across various agro-ecologies.
